# Intravenous iron and chronic obstructive pulmonary disease: a randomised controlled trial

**DOI:** 10.1136/bmjresp-2020-000577

**Published:** 2020-06-21

**Authors:** Peter Santer, Anne McGahey, Matthew C Frise, Nayia Petousi, Nick P Talbot, Richard Baskerville, Mona Bafadhel, Annabel H Nickol, Peter A Robbins

**Affiliations:** 1 Department of Physiology, Anatomy and Genetics, University of Oxford, Oxford, UK; 2 Oxford Respiratory Trials Unit, University of Oxford, Oxford, UK; 3 Oxford Centre for Respiratory Medicine, Oxford University Hospitals NHS Foundation Trust, Oxford, UK; 4 Nuffield Department of Medicine, University of Oxford, Oxford, UK; 5 Nuffield Department of Primary Care Health Sciences, University of Oxford, Oxford, UK

**Keywords:** COPD pathology, exercise, lung physiology

## Abstract

**Background:**

Increased iron availability modifies cardiorespiratory function in healthy volunteers and improves exercise capacity and quality of life in patients with heart failure or pulmonary hypertension. We hypothesised that intravenous iron would produce improvements in oxygenation, exercise capacity and quality of life in patients with chronic obstructive pulmonary disease (COPD).

**Methods:**

We performed a randomised, placebo-controlled, double-blind trial in 48 participants with COPD (mean±SD: age 69±8 years, haemoglobin 144.8±13.2 g/L, ferritin 97.1±70.0 µg/L, transferrin saturation 31.3%±15.2%; GOLD grades II–IV), each of whom received a single dose of intravenous ferric carboxymaltose (FCM; 15 mg/kg bodyweight) or saline placebo. The primary endpoint was peripheral oxygen saturation (SpO_2_) at rest after 1 week. The secondary endpoints included daily SpO_2_, overnight SpO_2_, exercise SpO_2_, 6 min walk distance, symptom and quality of life scores, serum iron indices, spirometry, echocardiographic measures, and exacerbation frequency.

**Results:**

SpO_2_ was unchanged 1 week after FCM administration (difference between groups 0.8%, 95% CI −0.2% to 1.7%). However, in secondary analyses, exercise capacity increased significantly after FCM administration, compared with placebo, with a mean difference in 6 min walk distance of 12.6 m (95% CI 1.6 to 23.5 m). Improvements of ≥40 m were observed in 29.2% of iron-treated and 0% of placebo-treated participants after 1 week (p=0.009). Modified MRC Dyspnoea Scale score was also significantly lower after FCM, and fewer participants reported scores ≥2 in the FCM group, compared with placebo (33.3% vs 66.7%, p=0.02). No significant differences were observed in other secondary endpoints. Adverse event rates were similar between groups, except for hypophosphataemia, which occurred more frequently after FCM (91.7% vs 8.3%, p<0.001).

**Conclusions:**

FCM did not improve oxygenation over 8 weeks in patients with COPD. However, this treatment was well tolerated and produced improvements in exercise capacity and functional limitation caused by breathlessness. These effects on secondary endpoints require confirmation in future studies.

**Trial registration number:**

ISRCTN09143837.

**Key messages**

- This study examines the question whether intravenous iron supplementation is well tolerated in patients with chronic obstructive pulmonary disease (COPD), and whether it leads to clinically significant improvements in oxygenation, exercise capacity or quality of life.
- In non-anaemic study participants with COPD, a single dose of intravenous ferric carboxymaltose had no effect on arterial oxygenation, but produced a reduction in breathlessness-related functional limitation and a small increase in exercise capacity that lasted for at least 8 weeks.
- If our findings are confirmed in future trials, intravenous ferric carboxymaltose could become an important therapeutic option in the management of patients with COPD.

## Introduction

In recent years, a striking relationship has been identified between iron availability and cardiorespiratory function that is not simply due to differences in haemoglobin concentration.^1 2^ In healthy volunteers, intravenous iron inhibits pulmonary vasoconstriction during hypoxia^3–5^ and reverses hypoxic pulmonary hypertension at high altitude.^6^ In patients with heart failure, iron supplementation increases exercise capacity and improves health-related quality of life,^1 7^ and similar findings have been reported in patients with idiopathic pulmonary hypertension.^8^ These effects may reflect the known interaction between iron and oxygen sensing at a cellular level.^2 9 10^

Chronic obstructive pulmonary disease (COPD) is characterised by progressive breathlessness and functional limitation, often accompanied by systemic hypoxia and pulmonary hypertension.^11 12^ The effects of iron status on cardiorespiratory function may be particularly significant in this setting, and in keeping with this, reduced iron availability in this patient group is associated with poor exercise tolerance and arterial hypoxaemia.^13–15^ Nevertheless, the effects of iron supplementation have not yet been studied in COPD.

We performed a preliminary randomised controlled clinical trial to test the hypothesis that intravenous ferric carboxymaltose (FCM) would lead to significant improvements in peripheral arterial oxygen saturation in patients with COPD, and to explore the effects on secondary outcomes including exacerbation frequency, exercise capacity and quality of life.

## Materials and methods

### Overview

This single-centre, randomised, placebo-controlled, double-blind trial investigated the effects of intravenous iron (FCM) in patients with COPD over an 8-week follow-up period. All participants provided written informed consent.

### Patient and public involvement

The trial design was informed by feedback from participants in previous studies involving intravenous iron infusions and by informal discussion with patients with COPD regarding the acceptability of study procedures.

### Participants

Between March 2015 and July 2017, we enrolled participants aged 18 years or older who had been diagnosed with COPD by a respiratory physician. All participants had a 1 s forced expiratory volume (FEV_1_) <80% predicted (ie, GOLD grades II–IV) and a smoking history of >15 pack years or another established cause of COPD, and had not experienced an exacerbation or a change in respiratory medication for at least 4 weeks prior to study onset. Iron deficiency was not a requirement for participation. Key exclusion criteria included iron overload, recent use of iron supplements, and significant renal or liver disease. A full list of inclusion and exclusion criteria is provided in the online supplementary material. Participants were recruited from research registries, primary and secondary care settings, and public outreach initiatives.

10.1136/bmjresp-2020-000577.supp1 Supplementary data

### Trial design and treatments

Eligible participants were randomly assigned in a 1:1 ratio to receive intravenous 15 mg/kg bodyweight FCM (Vifor Pharma UK, Bagshot Park, Surrey, UK; up to a maximum dose of 1000 mg) in 250 mL 0.9% saline, or a placebo infusion of 250 mL 0.9% saline. Randomisation was performed using the online Sealed Envelope randomisation system, which used the method of randomisation by minimisation with a 30% chance of simple random allocation. Participants were stratified according to oxygenation (resting peripheral oxygen saturation (SpO_2_) ≤94% vs >94%) and iron status (transferrin saturation <20% vs ≥20%). Investigators and participants were blinded to the treatment allocation, with infusions prepared and administered by research staff not involved in study-related assessments. The colour difference between infusions was concealed from the participants by covering the infusion equipment and the injection site with opaque drapes.

### Study procedures

Participants were screened for eligibility, which included medical review, venous blood sampling and pulmonary function testing. Participants were instructed on daily measurement and recording of oxygen saturation (observed over 2 min at rest) and peak expiratory flow (best of three attempts), as well as completion of a respiratory symptom diary card, which was used to determine the occurrence of exacerbations, as previously described.^16^ After 4 weeks of stability (ie, without exacerbations), participants attended a baseline and two follow-up visits at week 1 (±2 days) and week 8 (±5 days). Assessments at each visit included measurements of SpO_2_ at rest and during exercise (using a Pulsox-2 oximeter; Konica Minolta, Osaka, Japan), overnight pulse oximetry (using Pulsox-3i or Pulsox-300i oximeters; Konica Minolta), capillary blood gas analysis, venous blood sample analysis, 6 min walk test,^17^ prebronchodilator spirometry, symptom and quality of life questionnaires (COPD Assessment Test,^18^ St George’s Respiratory Questionnaire,^19^ modified Medical Research Council (MRC) Dyspnoea Scale,^20^ Dyspnoea-12 Score,^21^ Fatigue Severity Scale,^22^ Hospital Anxiety and Depression Scale,^23^ and Visual Analogue Scales). Transthoracic echocardiography was also performed to estimate pulmonary artery pressure.^3 5 6^ On completion of baseline assessments, participants received a single bolus infusion of the assigned study treatment through an intravenous cannula.

### Outcome measures

The primary outcome was the change in resting SpO_2_ between groups from baseline to 1 week after infusion. The secondary endpoints included changes from baseline to follow-up visits in oxygenation, 6 min walk distance (6MWD), laboratory parameters (iron status, haematological parameters, serum biochemistry, phosphate, hepatic and renal parameters, inflammatory markers), FEV_1_, symptom scores, BODE score,^24^ occurrence of exacerbations or adverse events, and exploratory echocardiographic measurements of trans-tricuspid pressure gradient.^3 5 6^

### Statistical analysis

Based on previous research,^15^ we estimated that a total sample size of 48 participants was needed to detect an absolute difference of 2.0%±2.5% (mean±SD) between groups in the change of oxygen saturation 1 week after infusion, with a statistical power of 80% and a significance level of 5% using an unpaired two-tailed Student’s t-test. Continuous data are reported as mean±SD or median (IQR), as appropriate. Comparisons of continuous outcome variables between groups at a single time point were made by Student’s t-test (normally distributed data) or Mann-Whitney U test (non-normal data). Categorical variables were analysed by χ^2^ test or Fisher’s exact test (if expected cell counts were less than 5). Linear mixed effects modelling was performed for longitudinal analysis of outcomes. For fixed effects, time (visit week) was used as a continuous covariate, with allocated group and status post iron infusion as factors. Correlation of residuals from repeated measures on participants was included in the model. All models included an intercept as a random effect. If exploratory data analysis suggested a curvilinear change over time, a quadratic time variable was introduced into the model. Statistical significance was assumed at p<0.05. Analysis was performed in IBM SPSS Statistics (V.24).

## Results

### Trial population

**Figure 1:**
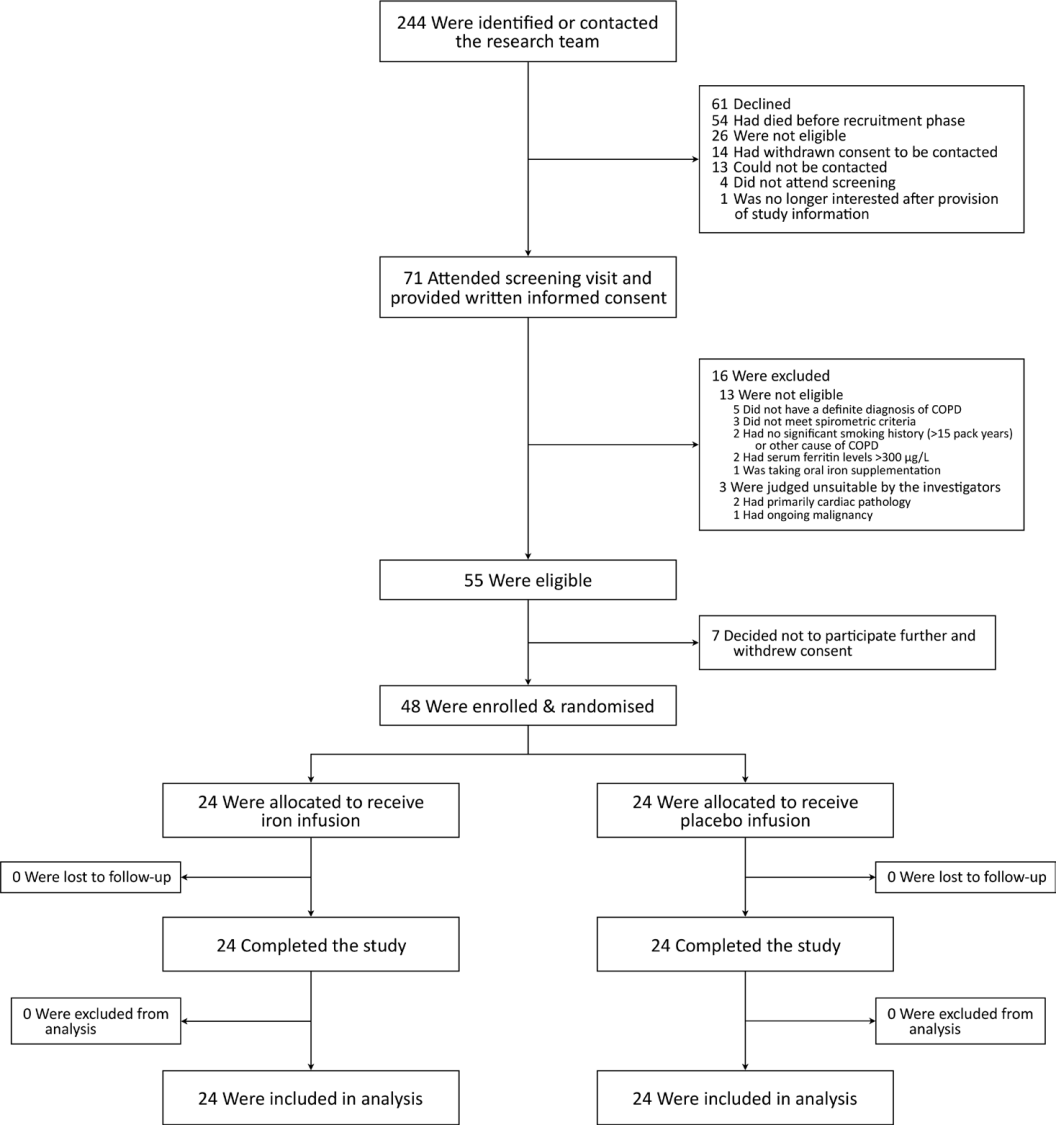
Participant flow diagram. COPD, chronic obstructive pulmonary disease.

A total of 244 participants were identified; 71 attended a screening visit, of whom 55 were eligible; 48 participants were enrolled and randomised. All enrolled participants completed the study and were included in the analysis (figure 1). Baseline characteristics were well matched between groups (table 1). One participant with no smoking history had alpha-1 antitrypsin deficiency. Comorbidities were primarily cardiovascular. The majority of participants (81.3%) had moderate to severe (GOLD II–III) airflow obstruction at baseline (table 1 and online supplementary table S1), and most (72.9%) were treated with inhaled triple therapy (long-acting muscarinic antagonist, long-acting beta agonist and inhaled corticosteroid; table 1).

**Table 1:** Demographic and baseline clinical characteristics of the participants

Characteristics	FCM (n=24)	Placebo (n=24)
Age, years	69.2±8.4	68.0±7.0
Sex, n (%)		
Male	15 (62.5)	19 (79.2)
Female	9 (37.5)	5 (20.8)
Body mass index, kg/m^2^	25.7±6.1	25.4±4.1
Smoking status, n (%)		
Former	18 (75.0)	16 (66.7)
Current	6 (25.0)	7 (29.2)
Never	0	1 (4.2)
Pack years	43 (31–67)	39 (28–67)^a^
Age at COPD onset, years	57.2±11.3^a^	58.2±5.3^b^
Age at smoking initiation, years	14.8±2.9	14.6±3.0^a^
Age at smoking cessation, years	59.0 ± 6.5^c^	57.5±8.7^d^
Exacerbations in previous year, n	1 (0–3)^a^	2 (1–3)^b^
Lung function*		
FEV_1_, L	1.16±0.50	1.35±0.38
FEV_1_, % of predicted	48.0±17.6	49.8±16.9
FEV_1_/FVC, %	44.8±9.0	40.4±10.2
GOLD grade, n (%)		
Mild (I)	–	–
Moderate (II)	9 (37.5)	10 (41.7)
Severe (III)	10 (41.7)	10 (41.7)
Very severe (IV)	5 (20.8)	4 (16.7)
Long-acting inhaled therapy, n (%)		
No long-acting treatment	1 (4)	1 (4)
LAMA only	4 (17)	2 (8)
LABA/LAMA	1 (4)	0 (0)
ICS/LABA	3 (13)	1 (4)
LAMA/ICS/LABA	15 (63)	20 (83)
Iron parameters		
Iron, µmol/L	16.1 (11.0–19.3)	15.2 (13.2–19.5)
Ferritin, µg/L	84.3 (65.1–110.6)	69.6 (38.5–151.9)
Transferrin, g/L	2.53±0.32	2.53±0.31
Transferrin saturation, %	28.0 (21.3–37.0)	26.5 (23.0–38.3)
Soluble transferrin receptor, nmol/L	16.2±4.4	17.1±5.0
Hepcidin, ng/mL	20.7 (12.7–29.1)	17.7 (8.0–25.1)
Haematological parameters†		
Haemoglobin, g/L	145.4±12.1	144.1±14.4
Mean corpuscular volume, fL	90.6±5.3	92.3±3.6
Mean cell haemoglobin, pg	29.9 (29.3–30.7)	30.4 (30.0–31.8)
Inflammatory parameters		
C reactive protein, mg/dL	2.4 (0.9–4.6)	3.7 (1.7–8.2)
Interleukin-6, pg/mL‡	4.48 (3.56–7.03)	5.87 (4.29–8.81)

Data are reported as mean±SD if normally distributed, or median (IQR) if non-normally distributed.

Missing data: ^a^n=23, ^b^n=22, ^c^n=18, ^d^n=16.

*Pulmonary function testing was performed at the screening visit unless results were available within 1 year prior to the visit.

†For haematological parameters: n=23 in both groups due to error in sample processing.

‡Interleukin-6 results are only reported for values above the assay detection threshold of 3.13 pg/mL (n=11 in the FCM group, n=16 in the placebo group).

COPD, chronic obstructive pulmonary disease; FCM, ferric carboxymaltose; FEV_1_, forced expiratory volume in 1 s; FVC, forced vital capacity; ICS, inhaled corticosteroid; LABA, long-acting beta agonist; LAMA, long-acting muscarinic antagonist.

Baseline iron, haematological and inflammation parameters were similar in the two groups (table 1 and online supplementary table S2). Using standard clinical criteria (ferritin <12 µg/L or transferrin saturation <16% or soluble transferrin receptor >28.1 nmol/L),^15 25^ 10% of participants were iron-deficient at baseline (three participants in the FCM group, two participants in the placebo group).

### Systemic iron status

There were substantial changes in serum iron indices following FCM. At 1 week, serum iron, ferritin, transferrin saturation and plasma hepcidin had all increased compared with placebo (all p<0.01; table 2), and serum transferrin and soluble transferrin receptor had fallen (both p<0.001). These effects on systemic iron status were still evident at 8 weeks. There was no effect of FCM on haemoglobin, but mean corpuscular volume and mean cell haemoglobin increased over the study period in the FCM-treated group, compared with placebo (p=0.003 and p<0.001, respectively). Other haematological indices were comparable between groups (online supplementary table S3).

**Table 2:** Temporal changes in laboratory parameters

Parameter	FCM (n=24)			Placebo (n=24)			Mean treatment effect of FCM (95% CI)	P value
	Baseline	Week 1	Week 8	Baseline	Week 1	Week 8		
Iron parameters								
Iron, µmol/L	16.5±7.3	28.8±9.5	17.3±5.4	16.8±6.1	16.5±6.7	15.3±5.3	4.6 (1.6 to 7.6)	0.003
Ferritin, µg/L	96.4±62.5	1149.5±282.2	395.2±161.4	97.7±78.1	91.4±70.6	95.8±79.5	318.8 (265.7 to 371.8)	<0.001
Transferrin, g/L	2.53±0.32	2.06±0.25	2.08±0.22	2.53±0.31	2.48±0.34	2.49±0.35	−0.47 (−0.53 to −0.40)	<0.001
Transferrin saturation, %	31.3±15.1	64.9±24.3	38.2±12.4	31.4±15.7	31.3±17.1	29.0±13.6	13.3 (7.8 to 18.7)	<0.001
Soluble transferrin receptor, nmol/L	16.2±4.4	13.0±3.5	14.3±3.4	17.1±5.0	16.8±5.2	16.8±5.7	−3.2 (−3.9 to −2.6)	<0.001
Hepcidin, ng/mL	21.8±12.2	106.8±33.9	58.5±22.5	21.8±18.9	18.7±17.3	26.7±25.6	39.4 (29.7 to 49.0)	<0.001
Haematological parameters								
Haemoglobin, g/L	145.4±12.1*	141.3±11.8*	144.5±12.3*	144.1±14.4*	140.3±12.9*	141.3±14.5*	0.4 (−3.2 to 4.0)	0.82
Mean corpuscular volume, fL	90.6±5.3*	91.3±5.2*	92.8±5.2*	92.3±3.6*	92.6±3.4*	92.2±3.7*	0.8 (0.3 to 1.3)	0.003
Mean cell haemoglobin, pg	30.2±2.2*	30.4±2.1*	30.7±1.9*	30.7±1.2*	30.4±1.1*	30.3±1.1*	0.7 (0.4 to 1.0)	<0.001
Inflammatory parameters								
C reactive protein, mg/dL	6.7±14.6	8.0±10.2	5.5±6.3	6.5±8.2	6.0±8.7	5.8±6.7	0.4 (−4.4 to 5.2)	0.86
Interleukin-6, pg/mL†	7.8±8.8 (13)	4.8±1.7 (11)	4.6±1.6 (11)	8.2±6.8 (8)	7.5±4.2 (14)	7.5±5.5 (6)	−3.4 (−7.6 to 0.8)	0.11
Serum phosphate, mmol/L	0.98±0.17	0.52±0.14	0.83±0.24	0.97±0.13	0.97±0.15	0.97±0.16	−0.46 (−0.51 to −0.40)	<0.001

All data are reported as mean±SD.

Statistical analysis was performed by linear mixed effects modelling.

P values and mean treatment effect are given for the fixed effect of ‘status post FCM infusion’.

*Descriptive data (mean±SD) are reported for participants with a valid measurement at each time point (n=23); cases with partially missing data were excluded. Participants with missing data were still included in the linear mixed effects model.

†Interleukin-6: values in parentheses indicate the number of data points with values below the assay’s detection threshold of 3.13 pg/mL. These values were not included in the descriptive statistics or the linear mixed effects model.

FCM, ferric carboxymaltose.

### Oxygenation

**Figure 2:**
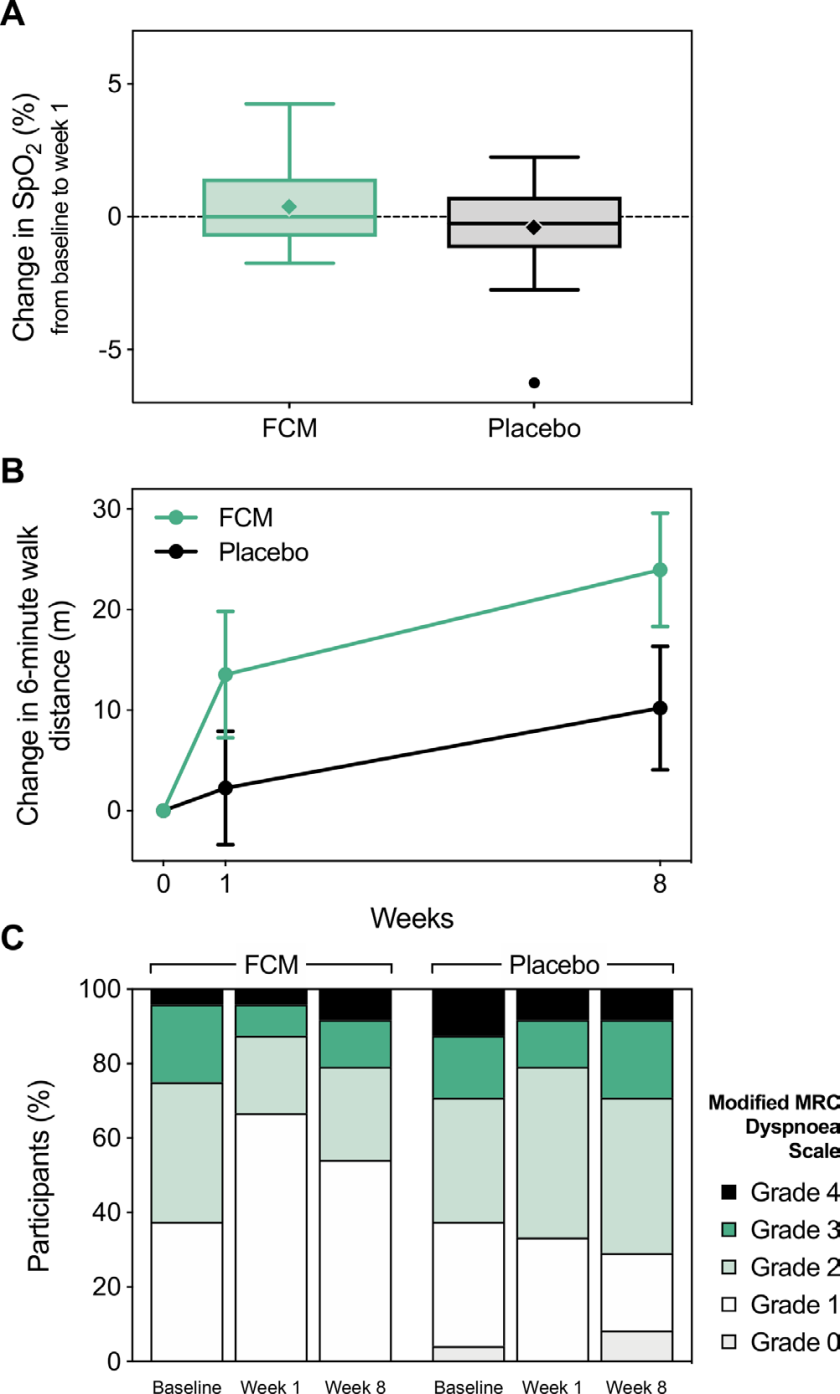
Primary and main secondary outcomes. (A) Box and whisker plot of the change in peripheral oxygen saturation from baseline to week 1 by treatment allocation (primary outcome). Solid lines indicate the median, boxes represent the IQR, whiskers extend to 1.5 times the IQR, diamonds (◆) represent the mean, and circles (●) represent outliers. (B) Relative changes in 6 min walk distance from baseline visit. Data are shown as mean±SE. Data from two participants in the placebo group were excluded from this graph due to missing 6 min walk tests at one study visit (n=22). (C) Proportions of modified MRC dyspnoea grades at each visit by treatment allocation. FCM, ferric carboxymaltose; MRC, Medical Research Council; SpO_2_, peripheral oxygen saturation.

At baseline, resting SpO_2_ was 94.1%±2.3% and 94.7%±1.6% in the FCM and placebo groups, respectively (table 3). Oxygen saturation changed by 0.4%±1.6% in the FCM group and −0.4%±1.7% in the placebo group after 1 week, resulting in a mean difference of 0.8% (95% CI −0.2% to 1.7%) between groups (figure 2A and table 3). Similarly, nocturnal pulse oximetry, capillary blood gas analysis (table 3 and online supplementary table S4) and daily self-measured oxygen saturations (online supplementary figure S1) remained unchanged after FCM.

**Table 3:** Primary and secondary outcomes

Parameter	FCM (n=24)			Placebo (n=24)			Mean treatment effect of FCM (95% CI)	P value
	Baseline	Week 1	Week 8	Baseline	Week 1	Week 8		
Oxygenation								
Resting SpO_2_, %	94.1±2.3	94.5±1.9	93.7±2.3	94.7±1.6	94.4±2.0	94.9±1.7	0.03 (−0.6 to 0.7)	0.93
Mean nocturnal SpO_2_, %	91.8±2.3^a^	92.3±1.9^a^	92.0±2.1^a^	92.0±2.2^b^	92.3±2.1^b^	92.2±2.5^b^	0.4 (−0.3 to 1.0)	0.32
Nocturnal SpO_2_ <90%, %	14.7±24.2^a^	14.6±18.8^a^	13.8±21.1^a^	16.4±25.6^b^	15.5±23.8^b^	15.8±24.6^b^	−0.3 (−8.1 to 7.6)	0.94
Oxygen desaturation index, per hour	8.0±9.2^a^	7.3±10.6^a^	7.8±13.2^a^	6.4±5.5^b^	6.7±5.7^b^	7.5±5.1^b^	−0.8 (−3.5 to 1.9)	0.56
Capillary PO_2_, kPa	9.35±1.13^b^	9.14±1.23^b^	9.19±1.07^b^	9.37±1.19^a^	9.27±1.11^a^	9.04±0.83^a^	−0.11 (−0.45 to 0.23)	0.52
Capillary SO_2_, %	94.6±2.0^b^	94.4±1.9^b^	94.4±1.6^b^	94.6±2.1^a^	94.4±1.6^a^	94.1±1.6^a^	−0.1 (−0.7 to 0.4)	0.64
6 min walk test								
Distance, m	330±89	343±93	354±92	335±92^b^	337±101^b^	345±104^b^	12.6 (1.6 to 23.5)	0.02
SpO_2_ change, %	−4.1±3.9	−4.1±3.8	−4.3±5.0	−5.6±6.3^b^	−5.0±5.7^b^	−5.2±6.6^b^	−0.1 (−1.7 to 1.5)	0.91
Heart rate change, per minute	18.4±16.8	14.0±15.8	19.3±17.6	17.7±14.3^b^	18.1±16.3^b^	17.5±17.8^b^	−2.5 (−9.8 to 4.9)	0.50
Symptom and quality of life scores*								
BODE score	3.5±2.1^a^	3.3±2.1^a^	3.4±2.1^a^	3.5±2.0^c^	3.5±2.0^c^	3.5±1.9^c^	−0.2 (−0.6 to 0.1)	0.22
COPD Assessment Test	14.5±6.3	14.8±7.8	16.6±7.3	16.5±7.1	15.5±8.0	18.3±7.5	0.03 (−1.6 to 1.6)	0.97
St George’s Respiratory Questionnaire total score	41.8±15.6	39.8±15.7	41.5±15.3	46.1±17.2	44.9±18.0	47.9±18.0	−2.3 (−4.8 to 0.1)	0.06
Modified MRC Dyspnoea Scale	1.9±0.9	1.5±0.8	1.8±1.0	2.0±1.1	2.0±0.9	2.0±1.1	−0.4 (−0.7 to −0.1)	0.008
Dyspnoea-12 Score	9.0±7.0	9.5±8.0	10.9±7.7	10.1±7.4	10.5±8.3	11.1±7.8	0.6 (−0.9 to 2.1)	0.43
Fatigue Severity Scale	33.0±15.7	31.0±13.7	32.0±12.6	39.8±16.0	37.6±16.8	41.1±15.2	−2.5 (−5.7 to 0.7)	0.13
Hospital Anxiety and Depression Scale-anxiety score	4.3±4.1	4.7±4.2	4.5±4.6	5.4±4.1	5.3±4.1	5.1±4.0	0.5 (−0.4 to 1.3)	0.31
Hospital Anxiety and Depression Scale-depression score	4.2±2.4	4.2±2.8	3.8±2.5	5.9±3.7	5.8±3.5	5.7±3.3	−0.1 (−0.7 to 0.6)	0.89
Visual Analogue Scales								
Dyspnoea	28.3±19.9	32.1±25.0	27.6±21.5	32.4±23.9	39.7±25.2	33.8±23.9	2.9 (−6.9 to 12.6)	0.56
Cough	28.6±25.4	34.4±26.5	34.6±27.5	31.3±28.0	30.6±26.0	29.9±22.7	6.1 (−4.3 to 16.5)	0.25
Sputum production	24.9±26.1	21.4±20.1	27.5±24.0	21.2±22.8	24.9±23.7	25.6±20.0	−2.6 (−10.1 to 4.8)	0.48
Sputum purulence	18.9±20.8^c^	17.1±19.4^c^	17.7±21.4^c^	24.4±27.0^c^	19.6±19.4^c^	26.0±20.7^c^	−2.9 (−10.2 to 4.4)	0.43
Spirometry								
FEV_1_, L	1.08±0.46^a^	1.04±0.44^a^	1.07±0.44^a^	1.25±0.34	1.27±0.39	1.27±0.38	−0.03 (−0.08 to 0.01)	0.12
FEV_1_, % of predicted	43.6±16.0^a^	42.3±15.2^a^	43.1±14.5^a^	46.0±15.2	46.3±16.0	46.5±16.5	−1.3 (−3.0 to 0.4)	0.13
FEV_1_/FVC, %	40.4±10.7^a^	41.3±10.7^a^	41.0±10.0^a^	39.8±10.9	39.2±11.1	39.3±10.4	1.1 (−0.8 to 3.0)	0.24
Echocardiography								
Tricuspid regurgitant jet measured, n (%)	8 (33.3)	8 (33.3)	6 (25.0)	7 (29.2)	7 (29.2)	6 (25.0)	–	–
Trans-tricuspid pressure gradient, mm Hg	28.8±4.5^d^	26.8±2.7^d^	28.2±2.4^d^	35.2±7.3^d^	35.8±7.6^d^	36.9±12.4^d^	−2.4 (−5.2 to 0.4)	0.09

All data are reported as mean±SD.

Statistical analysis was performed by linear mixed effects modelling.

P values and mean treatment effect are given for the fixed effect of ‘status post FCM infusion’.

Descriptive data (mean±SD) are reported for participants with a valid measurement at each time point (^a^n=23, ^b^n=22, ^c^n=21, ^d^n=6); cases with partially missing data were excluded. Cases with missing data were still included in the linear mixed effects model.

*Scale ranges: BODE 0–10, COPD Assessment Test 0–40, Dyspnoea-12 Score 0–36, Fatigue Severity Scale 9–63, Hospital Anxiety and Depression Scale 0–21, Likert scale 1–7, modified MRC scale 0–4, St George’s Respiratory Questionnaire 0–100, Visual Analogue Scales 0–100. For all scores a lower value is better.

COPD, chronic obstructive pulmonary disease; FCM, ferric carboxymaltose; FEV_1_, forced expiratory volume in 1 s; FVC, forced vital capacity; MRC, Medical Research Council; PO_2_, Partial pressure of oxygen; SO_2_, Oxygen saturation; SpO_2_, peripheral oxygen saturation.

### 6 min walk distance

By week 8, 6MWD had increased by 24±28 m in the FCM group, compared with 10±29 m in the placebo group (average treatment effect of FCM: 12.6 m, 95% CI 1.6 to 23.5 m; figure 2B and table 3). The proportion of participants achieving an increase of ≥40 m was significantly higher in the FCM group at week 1, compared with placebo (29.2% vs 0.0%, p=0.009). This effect was no longer statistically significant at week 8 (45.8% vs 27.3%, p=0.19). Changes in 6MWD for each individual participant are shown in online supplementary figure S4.

### Symptoms and quality of life

The modified MRC Dyspnoea Scale score fell significantly after FCM, but not after placebo infusion (average treatment effect: −0.4, 95% CI −0.7 to −0.1; table 3). At baseline, 62.5% of participants in both groups indicated significant breathlessness on exertion (MRC grade ≥2; figure 2C). By week 1, this percentage had fallen to 33.3% in the FCM group, but remained approximately constant at 66.7% in the placebo group (p=0.02). At week 8, the corresponding values were 45.8% in the FCM group and 70.8% in the placebo group (p=0.08). Other symptom and quality of life scores did not differ between groups (table 3).

### Other secondary outcomes

There was no effect of FCM on spirometric indices of airflow obstruction (table 3), and no effect on circulating inflammatory markers (C reactive protein and interleukin-6; table 2). Tricuspid regurgitation was detectable with echocardiography in 21 participants (43.8%; table 3), but was sufficient for estimation of the trans-tricuspid pressure gradient at all time points in only 12 participants (25%; six participants in each group). The pressure gradient appeared slightly lower after FCM infusion, but this reduction was not statistically significant (average treatment effect: −2.4 mm Hg, 95% CI −5.2 to 0.4 mm Hg; table 3).

### Exacerbations and adverse events

Seven participants in each group (29.2%) experienced exacerbations during the study period (table 4). Those in the FCM group had one exacerbation each, while those in the placebo group had a median (IQR) of 2 (1–2) events. The cumulative period of time (mean±SD) each participant spent with ongoing exacerbations was not different between groups (13±8 days in the FCM group, 10±6 days in the placebo group; difference: 3 days, 95% CI −6 to 12 days). Similarly, the time to first exacerbation was comparable between groups (23±12 days after randomisation in the FCM group and 18±21 days in the placebo group; difference: 5 days, 95% CI −15 to 24 days). The two groups made similar use of antibiotics, steroids and hospital facilities (table 4).

**Table 4:** Exacerbations and adverse events

	FCM (n=24)		Placebo (n=24)		P value
	Participants, n (%)	Events (n)	Participants, n (%)	Events (n)	
Exacerbations	7 (29.2)	7	7 (29.2)	12	1.00
Use of antibiotics	6 (25.0)	6	5 (20.8)	8	0.73
Use of steroids	5 (20.8)	5	5 (20.8)	6	1.00
Adverse events, all	22 (91.7)	48	17 (70.8)	28	0.14
Hypophosphataemia*	22 (91.7)	22	2 (8.3)	2	<0.001
Other abnormal laboratory tests†	8 (33.3)	10	7 (29.2)	8	0.76
Hypertension‡	5 (20.8)	5	4 (16.7)	4	1.00
Dyspnoea	3 (12.5)	3	0	0	0.23
Headache	2 (8.3)	2	4 (16.7)	4	0.67
Haematoma	2 (8.3)	2	2 (8.3)	2	1.00
Fever	1 (4.2)	1	0	0	1.00
Rash	1 (4.2)	1	0	0	1.00
Upper respiratory infection	1 (4.2)	1	0	0	1.00
Cough	1 (4.2)	1	0	0	1.00
Nasal congestion	0	0	1 (4.2)	1	1.00
Lung infection	0	0	1 (4.2)	2	1.00
Vomiting	0	0	1 (4.2)	1	1.00
Diarrhoea	0	0	1 (4.2)	1	1.00
Dyspepsia	0	0	1 (4.2)	1	1.00
Dizziness	0	0	1 (4.2)	1	1.00
Fall	0	0	1 (4.2)	1	1.00
Serious adverse events, all	0	0	2 (8.3)	2	0.49
Lung infection, requiring hospitalisation	0	0	1 (4.2)	1	1.00
Fall, requiring hospitalisation	0	0	1 (4.2)	1	1.00

Adverse events were recorded up to 1 week postinfusion and coded using CTCAE. Serious adverse events were recorded for the entire study duration. Pre-existing events were not considered adverse events unless they changed in severity (increase by one grade or more) or frequency. The total number of participants with adverse events does not correspond to the sum of the number of participants with a particular adverse event as some participants had multiple adverse events.

P values were calculated using χ^2^ or Fisher’s exact tests for differences in the number of participants with a given event.

*Hypophosphataemia was defined as phosphate levels <0.8 mmol/L. Phosphate levels were routinely measured in only 29 participants during the study (14 of those received FCM). Numbers reported here are based on the retrospective analysis of stored serum samples from all participants.

†These included, in order of frequency, elevated C reactive protein, hypoalbuminaemia, thrombocytosis, hypokalaemia, anaemia, hyperkalaemia, elevated white cell and neutrophil counts (no significant between-group differences for any of those values).

‡Hypertension was considered an adverse event if the elevated blood pressure was not pre-existent (ie, the grade of hypertension as defined by CTCAE observed immediately postinfusion or at week 1 was not present on any occasion prior to infusion) or was pre-existent but worsened (ie, an increase by at least one grade as defined by CTCAE immediately postinfusion or at week 1).

CTCAE, Common Terminology Criteria for Adverse Events; FCM, ferric carboxymaltose.

FCM administration was associated with a small rise in plasma alanine aminotransferase and alkaline phosphatase (online supplementary table S3), but neither parameter exceeded normal limits. Adverse events were comparable between groups (table 4), with the exception of hypophosphataemia. Phosphate levels fell below normal limits in the majority of participants after FCM (91.7%, compared with 8.3% in the placebo group, p<0.001), but all participants were asymptomatic and the levels recovered by week 8 (table 2). Five participants had a phosphate level of <0.3 mmol/L at week 1 and were prescribed phosphate substitution therapy, in line with local clinical guidelines.

Two adverse events requiring inpatient hospitalisation in the placebo group were reported as serious adverse events (table 4).

## Discussion

This study is the first clinical trial examining the effects of intravenous iron supplementation in patients with COPD. We found no impact of FCM on the primary outcome of oxygenation, but in common with clinical trials in patients with heart failure and idiopathic pulmonary hypertension, intravenous FCM produced statistically significant effects on exercise capacity and breathlessness-related functional limitation.

### Iron status in COPD

One working definition of iron deficiency is ferritin <12 µg/L or transferrin saturation <16% or soluble transferrin receptor >28.1 nmol/L.^15 25^ By this definition, 10% of our participants were iron-deficient. An alternative definition of iron deficiency that has been used in patients with heart failure and idiopathic pulmonary hypertension is ferritin <100 µg/L or ferritin <300 µg/L with transferrin saturation <20%.^7 26^ Using this definition, 69% of our participants would have been classified as iron-deficient. The contrast between these two percentages illustrates the difficulty of defining iron deficiency, particularly in the presence of inflammation.^25^ Additionally, it is clear that intravenous iron has biological effects even in the absence of iron deficiency, as evidenced by the major effect of intravenous iron administration on pulmonary vascular responses to hypoxia in healthy volunteers.^3–6^ These considerations informed our decision to recruit patients with or without iron deficiency in the current trial.

### Oxygenation

Iron has well-established effects on the pulmonary vascular responses to hypoxia.^2–6 10^ As such, it has been suggested that reduced iron availability may impair the matching of ventilation and perfusion in the lung and therefore reduce arterial oxygenation.^15 27^ This, coupled with a previous cross-sectional study that revealed an association between iron status and arterial hypoxaemia, informed our choice of arterial oxygenation as the primary outcome measure.^15^

The present study was powered to detect a 2% absolute rise in oxygen saturation after intravenous FCM. No such increase was identified in our study cohort. However, given that the mean oxygen saturation of the participants in this study at baseline (94.4%) was only marginally (<1%) lower than mean oxygen saturation for age-matched individuals from the general population,^28^ a significant improvement in oxygen saturation would have been very unlikely in our participants. It remains uncertain whether an effect of FCM on oxygen saturation would be detectable in a group of patients with more significant hypoxaemia at baseline.

### Mechanism of improvement in 6MWD

In patients with COPD, 6MWD correlates with the ability to perform activities of daily living and is inversely associated with mortality.^17 29^ Although the within-group increase of 24 m seen in the FCM group is less than the minimal clinically important difference (30–40 m) in this setting,^17 29^ it is strikingly similar to the improvement seen at 8 weeks in the landmark trial of intravenous FCM in patients with heart failure.^7^ In that study, exercise capacity rose progressively over at least 12 weeks, with a mean increase of ≥35 m seen 12 weeks and 24 weeks after FCM. It is therefore possible that a longer duration of follow-up might reveal a greater effect of intravenous iron.

The literature provides a number of possible explanations for the effect of FCM on exercise capacity in our study participants. First, the pulmonary artery pressure during exercise is reduced for at least 8 weeks after FCM in healthy older adults.^30^ While this was not measured in the current study, exercise-induced pulmonary hypertension is common in COPD and may limit exercise capacity.^12 31^

Second, in patients with heart failure, the benefits of intravenous iron may result from correction of cardiomyocyte iron deficiency,^9 32^ even in the presence of normal systemic iron status. Heart failure is a common comorbidity in patients with COPD,^33^ raising the possibility that similar mechanisms could underlie the positive effects of intravenous iron in the current study.

Finally, skeletal muscle dysfunction is increasingly recognised as an important contributor to exercise limitation and functional limitation in COPD,^34 35^ and muscle function depends crucially on the availability of iron, through its central role in oxidative phosphorylation. Increased skeletal muscle iron availability has also been suggested as an explanation for the benefits of iron in patients with heart failure,^35^ and in keeping with an effect on muscle oxidative capacity, iron administration increases the time to anaerobic threshold during submaximal exercise in patients with pulmonary hypertension.^8^

### MRC Dyspnoea Scale score

The improvement of MRC Dyspnoea Scale scores in the FCM group after 1 week is of particular interest, as lower scores are associated with increased survival.^36^ Furthermore, there are very few interventions in COPD that consistently improve this score.^37^ The disparity between the effect of FCM on the MRC scale and the lack of an effect on other symptom scores may arise because the former is not a direct measure of dyspnoea, but rather a function of the amount of physical activity required to induce breathlessness.^37^ Thus, the observed improvement in MRC score seems likely to reflect the improvement in 6MWD.

### Safety profile of FCM

In other patient groups, FCM has a very favourable safety profile.^38^ In the current study, there was no excess of adverse events in the FCM group, compared with placebo, with the exception of hypophosphataemia, which is a well-documented side effect of FCM.^38^ Importantly, given the theoretical concern that increased iron availability may facilitate the growth of bacteria in the lungs,^39^ we did not identify any significant increase in the rate or severity of infective exacerbations of COPD, although over a relatively short time course of the current trial. Future studies will be needed to confirm this finding over a longer period of time.

### Limitations

An important limitation of this preliminary trial is that the positive findings were limited to the secondary outcome measures. While this is of less concern in a first study to explore the potential benefits of intravenous iron in COPD, it nevertheless emphasises the need for further clinical trials in which an exercise-related variable is chosen as the primary outcome measure. This study is also a small single-centre study, which inevitably limits the degree to which its findings can be translated to a more general COPD population.

A further limitation of the current study is the lack of characterisation of the pulmonary vascular effects other than in a small subset of patients. Understanding the effects of intravenous iron on pulmonary artery pressures might have important implications for the targeting of iron therapy to specific populations of patients with COPD, for example those with known exercise-induced pulmonary hypertension or comorbid heart failure.

### Conclusions

In summary, in this preliminary randomised controlled trial, intravenous FCM had no effect on SpO_2_. It was, however, associated with small but significant improvements in exercise performance and functional status in non-anaemic patients with COPD. Intravenous iron is well tolerated, widely available and inexpensive. COPD is characterised by a very substantial symptom burden and a relative scarcity of effective interventions. If our findings are confirmed in future trials, intravenous iron could become an important therapeutic option in the management of this condition.
