# Supplementary material for: Intravenous iron and chronic obstructive pulmonary disease: a randomised controlled trial
Source: BMJ Open Respir Res. 2020 Jun 21;7(1):e000577. doi: 10.1136/bmjresp-2020-000577 (PMC7311010; doi:10.1136/bmjresp-2020-000577)
Supplement: Supplementary data [file bmjresp-2020-000577supp001.pdf]

**Online Supplement**

# Intravenous iron and chronic obstructive pulmonary disease: a randomised controlled trial

Peter Santer, Anne McGahey, Matthew Frise, Nayia Petousi, Nick Talbot, Richard Baskerville, Mona Bafadhel, Annabel Nickol, Peter Robbins

# Table of Contents

1. *Participant eligibility criteria* ........ *3*
Inclusion criteria ........ 3
Exclusion criteria ........ 3
2. *Study design* ........ *4*
3. *Subgroup analyses* ........ *5*
4. *Tables and Figures* ........ *7*
Figure S1. Self-recorded daily peripheral oxygen saturation measurements ........ 7
Figure S2. Self-recorded daily peak expiratory flow rate measurements ........ 8
Figure S3. Primary and key secondary outcomes in iron-deficient and iron-replete participants ... 9
Figure S4. Changes in 6-minute walk distance for individual study participants ........ 10
Table S1. Pulmonary function test results obtained at the screening visit ........ 11
Table S2. Additional baseline laboratory parameters ........ 13
Table S3. Temporal changes in additional laboratory parameters ........ 14
Table S4. Capillary blood gas analysis ........ 15
5. *References* ........ *16*

# 1. Participant eligibility criteria

## Inclusion criteria

- Participants with a diagnosis of COPD, with at least moderate disease (grades II–IV of the GOLD criteria classification[1], i.e. $FEV_1$ < 80% predicted and $FEV_1$/FVC < 70%)
- Significant smoking history (i.e. > 15 pack years) or other definite cause of COPD
- Stable COPD for at least four weeks at study initiation (i.e. absence of exacerbation and no changes in respiratory medication)
- Able (in the investigator's opinion) and willing to comply with all study requirements
- Willing and able to give informed consent for participation in the study
- Male or female, aged 18 years or older

## Exclusion criteria

- Female participants who are pregnant, lactating, planning pregnancy during the course of the study or of childbearing potential unless using effective contraception for the duration of the study
- Participants taking iron supplements (in the six weeks prior to study initiation) or who have had a blood transfusion (in the six months prior to study initiation)
- Iron overload, defined as ferritin > 300 µg/L
- Hypersensitivity to previous iron infusion
- Evidence of bacteremia or respiratory infection
- Significant renal or liver disease (as judged by the investigator)

## 2. Study design

The original study design, as first published in the clinical trials registration (ISRCTN 09143837), aimed at determining whether intravenous iron attenuated the pulmonary arterial systolic pressure rise (PASP) with a long hypoxic exposure in COPD immediately following an infusion of iron compared to placebo. This study design was based on previous literature showing beneficial effects of intravenous iron in ameliorating pulmonary arterial systolic pressure increases during hypoxia in healthy volunteers.

This approach was subsequently fount to not be feasible and recruiting the necessary number of participants would not have been possible due to several limitations: It was determined, that exposing already hypoxic patients with chronic pulmonary disease to prolonged hypoxia was unlikely to be feasible in a large number of patients. Furthermore, preliminary attempts at measuring PASP in patients with COPD were complicated by the limitations of echocardiographic assessment of pulmonary pressure in these patients. Echocardiograms of sufficient quality could only be obtained in a small fraction of patients. Therefore, this approach would not have been feasible as a primary endpoint.

Given these limitations, the study protocol was changed to focus on a surrogate endpoint (peripheral oxygen saturation), which was supported by retrospective research.[2] The study protocol and registration were amended accordingly.



Santer P, *et al. BMJ Open Resp Res* 2020; 7:e000577. doi: 10.1136/bmjresp-2020-000577

## 3. Subgroup analyses

As suggested in the review process, we report subgroup analyses for the primary (peripheral oxygen saturation) and key secondary outcomes (6-minute walk distance and modified MRC score) in iron-deficient and iron-replete participants, using a common definition of iron deficiency (ferritin < 100 µg/L or ferritin 100–299 µg/L with transferrin saturation < 20%).[3] Using this definition, 33 (68.8%) participants in our study fulfilled the criteria for iron deficiency. In the iron-deficient subgroup, 16 participants received FCM and 17 received placebo; in the iron-replete subgroup, 8 participants received FCM and 7 received placebo.

*Peripheral oxygen saturation*: No significant differences in change of $SpO_2$ from baseline to week 1 between treatments were observed in iron-deficient and iron-replete participants. In the iron-deficient group, the change (mean ± SD) in $SpO_2$ from baseline to week 1 was 0.6 ± 1.5% in the FCM group and −0.4 ± 1.8% in the placebo group (difference: 1.0%, 95% CI −0.2 to 2.2%, P = 0.102). In the iron-replete subgroup, $SpO_2$ changed by 0.0 ± 1.8% in patients receiving FCM, and by −0.3 ± 1.6% in patients receiving placebo (difference: 0.3%, 95% CI −1.6 to 2.2, P = 0.723, Figure S3, panel A).

*Six-minute walk distance (6MWD)*: Administration of FCM resulted in increased 6MWD in both iron-deficient and iron-replete participants. In iron-deficient participants, 6MWD increased by 19.7 ± 25.3 m from baseline to week 8 after FCM administration and by 6.9 ± 26.1 m after placebo (difference: 12.8 m, 95% CI −5.7 to 31.4 m, P = 0.169). In iron-replete participants, the change from baseline to week 8 was 32.5 ± 32.1 m after FCM and 19.2 ± 36.1 m after placebo, respectively (difference: 13.3 m, 95% CI −26.5 to 53.1 m, P = 0.479). In a linear mixed effects model, the average effect of FCM on 6MWD was 14.0 m (95% CI 2.2 to 25.9 m, P = 0.021) in iron-deficient participants and 8.6 m (95% CI −14.9 to 32.1 m, P = 0.458) in iron-replete participants (Figure S3, panel B).



Santer P, *et al. BMJ Open Resp Res* 2020; 7:e000577. doi: 10.1136/bmjresp-2020-000577

*Modified MRC score*: In iron-deficient participants, mMRC scores at week 1 changed by −0.4 ± 0.8 from baseline after FCM, compared to 0.2 ± 0.5 after placebo administration (difference: −0.6, 95% CI −1.0 to −0.1, P = 0.029). In iron-replete participants, mMRC scores were reduced from baseline after both FCM (−0.5 ± 0.5) and placebo (−0.6 ± 0.5) with a difference of 0.1 (95% CI −0.5 to 0.7%, P = 0.800). In a linear mixed effect model, the average effect of FCM on mMRC was −0.35 (95% CI −0.71 to 0.00, P = 0.05) in iron-deficient participants and −0.42 (95% CI −0.87 to 0.03, P = 0.064) in iron-replete participants (Figure S3, panel C).

## 4. Tables and Figures

### Figure S1. Self-recorded daily peripheral oxygen saturation measurements

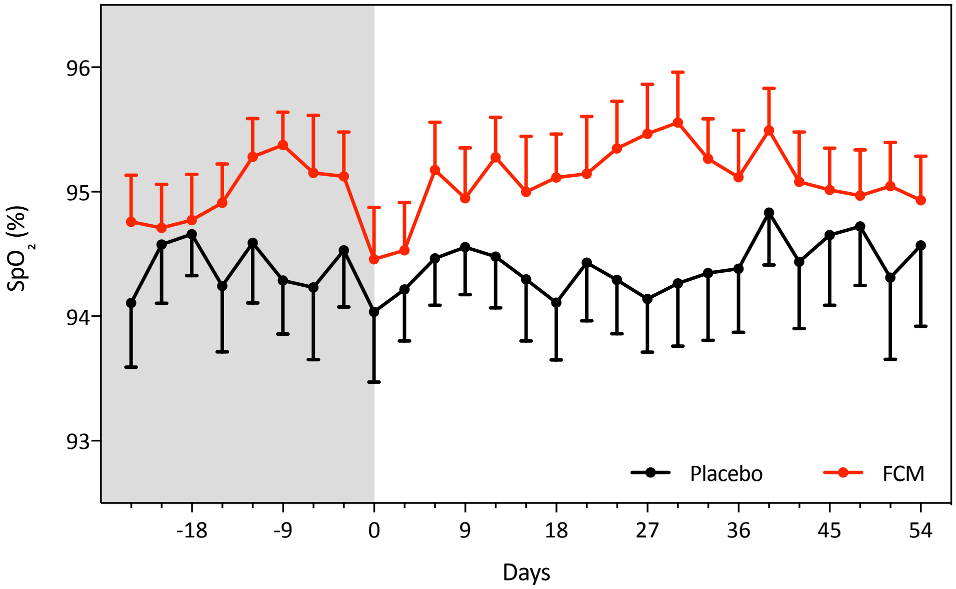


Participants measured and recorded their peripheral oxygen saturation daily, starting at the screening visit. Day 0 marked the baseline visit. Self-measured oxygen saturations did not differ significantly between the two groups (P = 0.170 for the effect of iron administration). Data points represent the average of the three preceding days. Statistical comparison was performed by linear mixed effects modelling as described in the main manuscript; pre-baseline data (shaded area) were not included in the analysis. All data are expressed as mean ± standard error. FCM: ferric carboxymaltose.



Santer P, *et al. BMJ Open Resp Res* 2020; 7:e000577. doi: 10.1136/bmjresp-2020-000577

## Figure S2. Self-recorded daily peak expiratory flow rate measurements

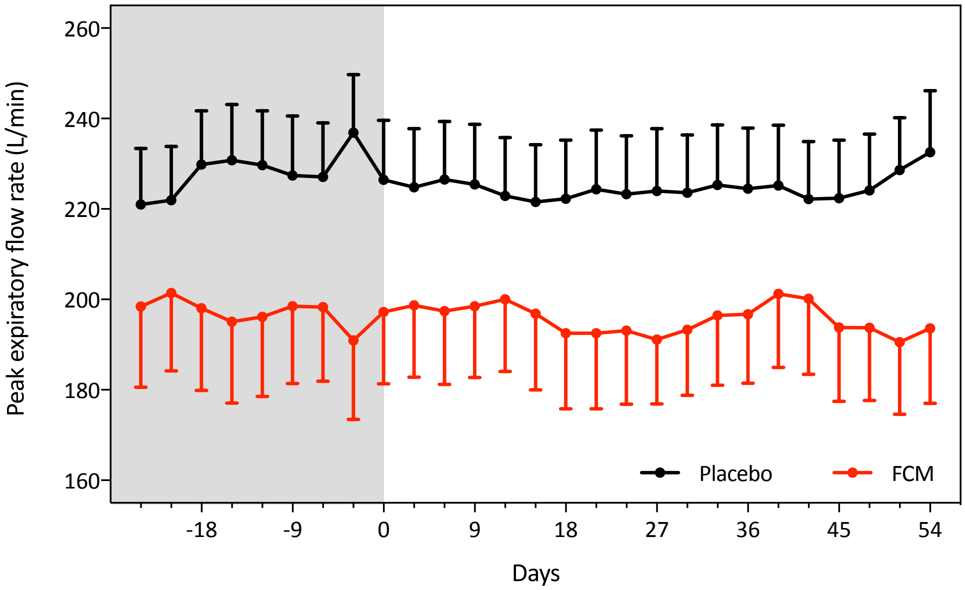


Participants measured and recorded their peak expiratory flow rates (best out of 3 attempts) daily, starting at the screening visit. Day 0 marked the baseline visit. Peak expiratory flow rates did not differ significantly between the two groups (P = 0.765 for the effect of iron administration). Data points represent the average of the three preceding days. Statistical comparison was performed by linear mixed effects modelling as described in the main manuscript; pre-baseline data (shaded area) were not included in the analysis. All data are expressed as mean ± standard error. FCM: ferric carboxymaltose.



Santer P, *et al. BMJ Open Resp Res* 2020; 7:e000577. doi: 10.1136/bmjresp-2020-000577

Figure S3. Primary and key secondary outcomes in iron-deficient and iron-replete participants

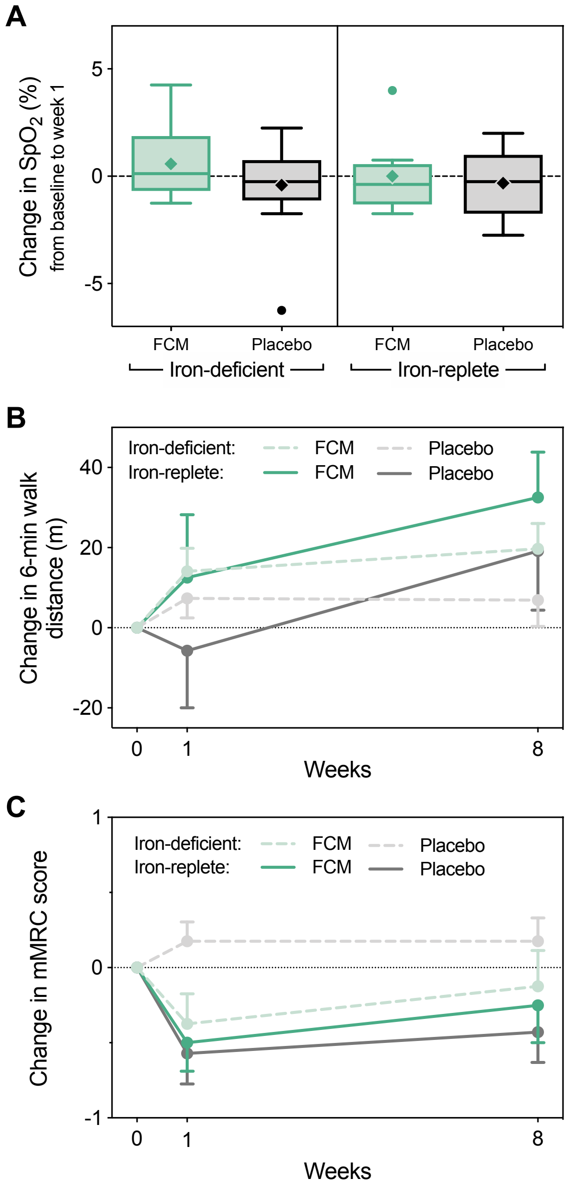


Primary (A) and key secondary endpoints (B and C) are shown for subgroups of iron-deficient and iron-replete participants. All data are expressed as mean ± standard error. FCM: ferric carboxymaltose.



Santer P, *et al. BMJ Open Resp Res* 2020; 7:e000577. doi: 10.1136/bmjresp-2020-000577

Figure S4. Changes in 6-minute walk distance for individual study participants

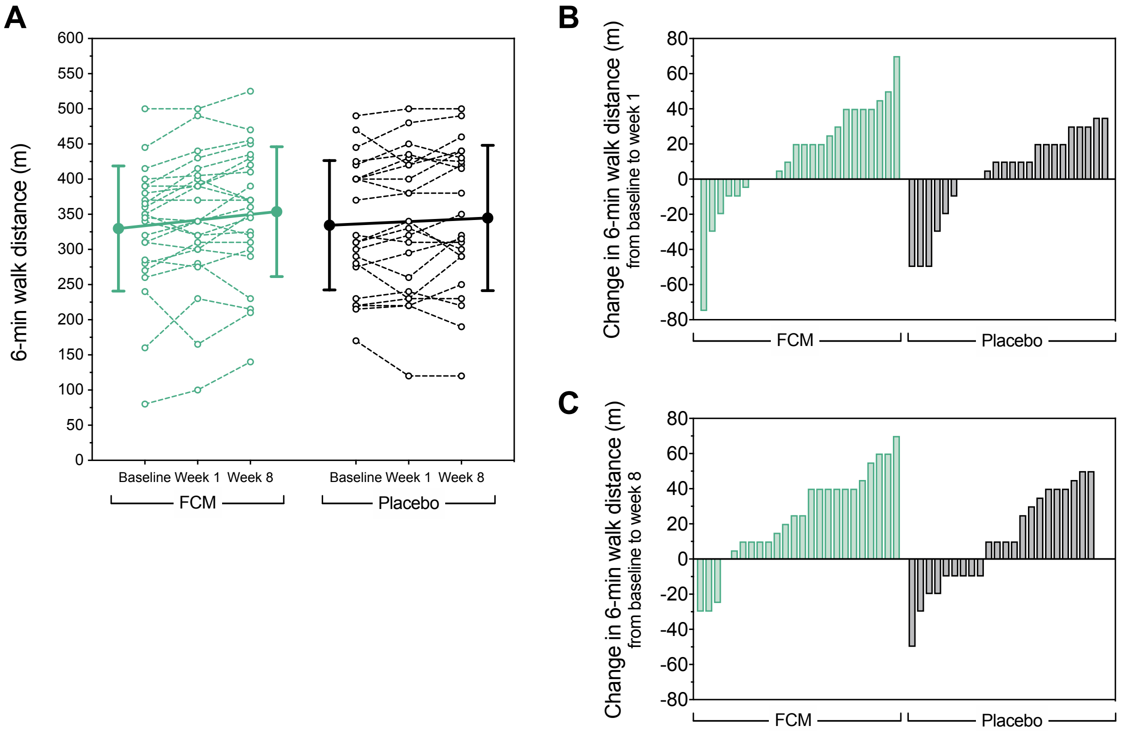


Six-minute walk distances are shown at baseline, week 1 and week 8 for each individual study participant (A, dashed lines); solid lines represents the change in mean walk distance from baseline to week 8 (error bars represent standard deviations). Relative changes in 6-minute walk distance from baseline to week 1 (B) and baseline to week 8 (C) are shown for each individual study participant. FCM: ferric carboxymaltose.



Santer P, *et al. BMJ Open Resp Res* 2020; 7:e000577. doi: 10.1136/bmjresp-2020-000577

## Table S1. Pulmonary function test results obtained at the screening visit

| **Parameter**, unit | | **FCM** (n = 24) | **Placebo** (n = 24) | **P value** |
|---|---|---|---|---|
| **Peak expiratory flow rate (PEFR)** | *Measured, L min$^{-1}$* | 246.6 ± 92.5 | 277.6 ± 74.1 | 0.205 |
| | *% predicted* | 60.3 ± 19.0 | 63.9 ± 18.8 | 0.518 |
| **Forced expiratory volume in one second ($FEV_1$)** | *Measured, L* | 1.16 ± 0.50 | 1.35 ± 0.38 | 0.162 |
| | *% predicted* | 48.0 ± 17.6 | 49.8 ± 16.9 | 0.714 |
| **Forced vital capacity (FVC)** | *Measured, L* | 2.56 ± 0.79 | 3.37 ± 0.84 | **0.001** |
| | *% predicted* | 83.0 ± 21.3 | 95.2 ± 19.9 | **0.044** |
| **Vital capacity (VC)** | *Measured, L* | 2.74 ± 0.84 | 3.48 ± 0.83 [a] | **0.004** |
| | *% predicted* | 88.1 ± 22.5 | 96.8 ± 17.4 [a] | 0.145 |
| **$FEV_1$/VC ratio** | *Measured, %* | 43.2 ± 9.9 | 39.1 ± 10.8 [a] | 0.177 |
| **$FEV_1$/FVC ratio** | *Measured, %* | 44.8 ± 9.0 | 40.4 ± 10.2 | 0.116 |
| **Functional residual capacity (FRC)** | *Measured, L* | 5.11 ± 1.54 [b] | 5.01 ± 1.50 [c] | 0.838 |
| | *% predicted* | 156.5 (133.2–174.7) [b] | 147.8 (123.7–175.2) [c] | 0.616 |
| **Residual volume (RV)** | *Measured, L* | 4.59 ± 1.51 [b] | 3.93 ± 1.27 [c] | 0.169 |
| | *% predicted* | 191.0 (163.2–219.0) [b] | 168.4 (120.0–198.5) [c] | 0.107 |
| **Total lung capacity (TLC)** | *Measured, L* | 7.43 ± 1.50 [b] | 7.59 ± 1.88 [c] | 0.773 |
| | *% predicted* | 123.5 (118.7–133.2) [b] | 124.8 (107.4–133.7) [c] | 0.639 |
| **RV/TLC ratio** | *Measured, %* | 61.1 ± 10.8 [b] | 51.1 ± 6.9 [c] | **0.002** |



Santer P, *et al. BMJ Open Resp Res* 2020; 7:e000577. doi: 10.1136/bmjresp-2020-000577

| **Parameter**, unit | | **FCM** (n = 24) | **Placebo** (n = 24) | **P value** |
|---|---|---|---|---|
| **Airway resistance ($R_{aw}$)** | *Measured, kPa $L^{-1}$ $s^{-1}$* | 0.42 (0.32–0.56) [b] | 0.43 (0.32–0.56) [c] | 0.802 |
| **Specific airway conductance ($sG_{aw}$)** | *Measured, $kPa^{-1}$ $s^{-1}$* | 0.50 (0.27–0.61) [b] | 0.45 (0.39–0.55) [d] | 0.545 |
| | *% predicted* | 52.0 (24.4–82.3) [d] | 50.5 (39.7–56.7) [e] | 0.886 |
| **Alveolar volume (VA)** | *Measured, L* | 4.24 ± 1.18 [a] | 5.06 ± 1.22 [a] | **0.025** |
| | *% predicted* | 74.8 ± 14.0 [a] | 81.8 ± 13.0 [a] | 0.086 |
| **Transfer factor for carbon monoxide ($T_{LCO}$)** | *Measured, mmol $min^{-1}$ $kPa^{-1}$* | 3.80 ± 1.13 [a] | 3.80 ± 1.28 [a] | 0.995 |
| | *% predicted* | 50.1 ± 14.2 [a] | 46.9 ± 15.3 [a] | 0.467 |
| **Transfer coefficient for carbon monoxide ($K_{CO}$)** | *Measured, mmol $min^{-1}$ $kPa^{-1}$ $L^{-1}$* | 0.92 ± 0.22 [a] | 0.78 ± 0.27 [a] | 0.057 |
| | *% predicted* | 67.6 ± 18.8 [a] | 57.5 ± 15.2 [a] | 0.051 |

*Pulmonary function tests were performed on all study participants at the screening visit unless results from spirometry or a full pulmonary function tests were available within one year prior to the screening visit. Data are reported as mean ± standard deviation, if normally distributed, or median (interquartile range), if not normally distributed. Statistical analysis was performed by independent samples Student t-test (normally distributed data) or Mann-Whitney U test (non-normally distributed data). Missing data: [a] n = 23, [b] n = 19, [c] n = 17, [d] n = 16, [e] n = 14. FCM: ferric carboxymaltose.*



Santer P, *et al. BMJ Open Resp Res* 2020; 7:e000577. doi: 10.1136/bmjresp-2020-000577

## Table S2. Additional baseline laboratory parameters

| **Parameter**, unit | **FCM** (n = 24) | **Placebo** (n = 24) | **P value** |
|---|---|---|---|
| **Hematological markers** | | | |
| Haematocrit, % | 43.6 ± 3.1 [a] | 43.3 ± 3.8 [a] | 0.778 |
| White cell count, x $10^9$/L | 6.86 (6.36–8.08) [a] | 8.14 (6.86–11.28) [a] | **0.039** |
| Neutrophil count, x $10^9$/L | 4.42 (4.03–5.55) [a] | 5.40 (4.52–7.86) [a] | **0.023** |
| Eosinophil count, x $10^9$/L | 0.18 ± 0.10 [a] | 0.19 ± 0.14 [a] | 0.739 |
| Platelet count, x $10^9$/L | 255 (227–278) [a] | 261 (221–323) [a] | 0.345 |
| **Serum biochemistry** | | | |
| Sodium, mmol/L | 138.4 ± 2.0 | 138.6 ± 3.8 | 0.848 |
| Potassium, mmol/L | 4.1 ± 0.4 | 4.2 ± 0.4 | 0.392 |
| Phosphate, mmol/L | 0.98 ± 0.17 | 0.97 ± 0.13 | 0.656 |
| Urea, mmol/L | 5.1 ± 1.9 | 6.2 ± 1.8 | **0.041** |
| Creatinine, µmol/L | 67.5 ± 14.9 | 79.3 ± 18.0 | **0.018** |
| Glucose, mmol/L | 5.5 ± 1.0 [b] | 5.6 ± 1.2 [b] | 0.805 |
| Bilirubin, µmol/L | 9.0 (6.3–11.5) | 8.0 (6.0–15.0) | 0.942 |
| Alanine aminotransferase, IU/L | 18.4 ± 6.6 | 19.5 ± 8.5 | 0.611 |
| Alkaline Phosphatase, IU/L | 72.4 ± 13.5 | 77.7 ± 25.3 | 0.366 |
| Albumin, g/L | 36.7 ± 2.8 | 36.4 ± 2.6 | 0.752 |

*Data are reported as mean ± standard deviation, if normally distributed, or median (interquartile range), if not normally distributed. Statistical analysis was performed by independent samples Student t-test (normally distributed data) or Mann-Whitney U test (non-normally distributed data). Missing data: [a] n = 23, [b] n = 22. FCM: ferric carboxy-maltose.*

## Table S3. Temporal changes in additional laboratory parameters

| **Parameter**, unit | **FCM** (n = 24) | | | **Placebo** (n = 24) | | | **P value** |
|---|---|---|---|---|---|---|---|
| | **Baseline** | **Week 1** | **Week 8** | **Baseline** | **Week 1** | **Week 8** | |
| **Hematological markers** | | | | | | | |
| Hematocrit, % | 43.6 ± 0.6 [a] | 42.5 ± 0.7 [a] | 43.6 ± 0.7 [a] | 43.3 ± 0.8 [a] | 42.7 ± 0.9 [a] | 43.0 ± 0.9 [a] | 0.478 |
| Erythropoietin, mIU/mL | 8.1 ± 0.7 | 7.8 ± 0.6 | 7.6 ± 0.8 | 9.9 ± 0.9 | 9.8 ± 1.3 | 9.8 ± 1.2 | 0.540 |
| White cell count, x $10^9$/L | 7.7 ± 0.6 [a] | 7.2 ± 0.6 [a] | 7.6 ± 0.5 [a] | 9.0 ± 0.6 [a] | 8.4 ± 0.6 [a] | 9.0 ± 0.4 [a] | 0.152 |
| Neutrophil count, x $10^9$/L | 5.2 ± 0.6 [a] | 4.8 ± 0.5 [a] | 5.0 ± 0.5 [a] | 6.3 ± 0.5 [a] | 5.7 ± 0.5 [a] | 6.3 ± 0.4 [a] | 0.167 |
| Eosinophil count, x $10^9$/L | 0.18 ± 0.02 [a] | 0.18 ± 0.02 [a] | 0.19 ± 0.02 [a] | 0.19 ± 0.03 [a] | 0.19 ± 0.02 [a] | 0.17 ± 0.03 [a] | 0.689 |
| Platelet count, x $10^9$/L | 261 ± 11 [a] | 254 ± 14 [a] | 270 ± 17 [a] | 304 ± 30 [a] | 296 ± 27 [a] | 290 ± 24 [a] | 0.294 |
| **Serum biochemistry** | | | | | | | |
| Sodium, mmol/L | 138.4 ± 0.4 | 138.3 ± 0.5 | 138.5 ± 0.4 | 138.6 ± 0.8 | 138.4 ± 0.5 | 138.1 ± 0.5 | 0.870 |
| Potassium, mmol/L | 4.1 ± 0.1 | 4.0 ± 0.1 | 3.9 ± 0.1 | 4.2 ± 0.1 | 4.1 ± 0.1 | 4.2 ± 0.1 | 0.229 |
| Urea, mmol/L | 5.1 ± 0.4 | 4.8 ± 0.3 | 5.1 ± 0.4 | 6.2 ± 0.4 | 6.1 ± 0.4 | 6.3 ± 0.4 | 0.084 |
| Creatinine, µmol/L | 67.5 ± 3.1 | 65.0 ± 3.2 | 67.3 ± 2.9 | 79.3 ± 3.7 | 78.8 ± 3.4 | 80.0 ± 3.9 | **0.005** |
| Glucose, mmol/L | 5.5 ± 0.2 [b] | 5.8 ± 0.4 [b] | 5.6 ± 0.4 [b] | 5.6 ± 0.3 [b] | 5.4 ± 0.3 [b] | 5.4 ± 0.2 [b] | 0.238 |
| Bilirubin, µmol/L | 9.1 ± 0.6 | 8.1 ± 0.5 | 9.0 ± 0.7 | 10.5 ± 1.3 | 10.2 ± 1.2 | 10.0 ± 1.1 | 0.064 |
| Alanine aminotransferase, IU/L | 18.4 ± 1.3 | 23.6 ± 1.9 | 19.3 ± 1.4 | 19.5 ± 1.7 | 18.5 ± 1.7 | 20.1 ± 1.7 | **0.001** |
| Alkaline Phosphatase, IU/L | 72.4 ± 2.7 | 74.6 ± 2.8 | 81.4 ± 3.0 | 77.7 ± 5.2 | 75.0 ± 5.3 | 76.7 ± 5.4 | **0.005** |
| Albumin, g/L | 36.7 ± 0.6 | 35.6 ± 0.5 | 36.0 ± 0.6 | 36.4 ± 0.5 | 35.3 ± 0.6 | 35.3 ± 0.6 | 0.880 |

*[a, b] Results are reported only for participants with a valid data point at each visit ([a] n = 23, [b] n = 22); cases with partially missing data were excluded from this table, but not from the statistical model. All data are reported as mean ± standard error. Statistical analysis was performed by linear mixed effects modelling; p values are reported for the fixed effect of "status post FCM infusion". FCM: ferric carboxymaltose.*



Santer P, *et al. BMJ Open Resp Res* 2020; 7:e000577. doi: 10.1136/bmjresp-2020-000577

## Table S4. Capillary blood gas analysis

| **Parameter**, unit | **FCM** [a] (n = 24) | | | **Placebo** [b] (n = 24) | | | **P value** |
|---|---|---|---|---|---|---|---|
| | Baseline | Week 1 | Week 8 | Baseline | Week 1 | Week 8 | |
| **pH** | 7.45 ± 0.01 | 7.45 ± 0.00 | 7.45 ± 0.00 | 7.45 ± 0.01 | 7.45 ± 0.01 | 7.44 ± 0.01 | 0.527 |
| **Partial pressure of $CO_2$ ($PCO_2$)**, kPa | 4.85 ± 0.10 | 4.95 ± 0.11 | 4.99 ± 0.08 | 4.76 ± 0.09 | 4.81 ± 0.10 | 5.00 ± 0.13 | 0.968 |
| **Partial pressure of $O_2$ ($PO_2$)**, kPa | 9.35 ± 0.24 | 9.14 ± 0.26 | 9.19 ± 0.23 | 9.37 ± 0.25 | 9.27 ± 0.23 | 9.04 ± 0.17 | 0.519 |
| **Oxygen saturation ($SO_2$)**, % | 94.6 ± 0.4 | 94.4 ± 0.4 | 94.4 ± 0.4 | 94.6 ± 0.4 | 94.4 ± 0.3 | 94.1 ± 0.3 | 0.640 |
| **Base Excess**, mmol/L | 1.31 ± 0.43 | 1.62 ± 0.33 | 2.11 ± 0.36 | 0.92 ± 0.43 | 1.00 ± 0.44 | 1.40 ± 0.49 | 0.378 |
| **Bicarbonate ($HCO_3$)**, mmol/L | 25.5 ± 0.4 | 25.8 ± 0.3 | 26.2 ± 0.3 | 25.2 ± 0.4 | 25.2 ± 0.4 | 25.6 ± 0.4 | 0.441 |

*[a, b] Results are reported only for participants with a valid data point at each visit ([a] n = 22, [b] n = 23); cases with partially missing data were excluded from this table, but not from the statistical model. All data are reported as mean ± standard error. Statistical analysis was performed by linear mixed effects modelling; p values are reported for the fixed effect of "status post FCM infusion". FCM: ferric carboxymaltose.*



Santer P, *et al. BMJ Open Resp Res* 2020; 7:e000577. doi: 10.1136/bmjresp-2020-000577

## 5. References


1. Vogelmeier CF, Criner GJ, Martinez FJ, et al. Global Strategy for the Diagnosis, Management, and Prevention of Chronic Obstructive Lung Disease 2017 Report. GOLD Executive Summary. Am J Respir Crit Care Med 2017;195:557-82.

2. Nickol AH, Frise MC, Cheng HY, et al. A cross-sectional study of the prevalence and associations of iron deficiency in a cohort of patients with chronic obstructive pulmonary disease. BMJ Open 2015;5:e007911.

3. Anker SD, Comin Colet J, Filippatos G, et al. Ferric carboxymaltose in patients with heart failure and iron deficiency. N Engl J Med 2009;361:2436-48.
